# Book Review

**Published:** 1915-12

**Authors:** 


### BOOK REVIEWS

DISEASES OF THE SKIN, by Henry H. Hazen, A.B., M.D., Professor of Dermatology in the Medical Department of George town University; Professor of Dermatology in the Medical Department of Howard University; Sometime Assistant in Dermatology in the Johns Hopkins University; Member of the American Dermatological Association. 539 pages. 233 Illustrations, including 4 color plates. St. Louis, C. V. Mosby Company, 1915. Cloth, $4.00.

The book is planned to present the practical matters in the every-day treatment of diseases of the skin first and then to take up such of the extraordinary and rare diseases as there is space for in a convenient volume. The style of the author deserves the commendation for its clarity and his plan invites one to read the book and become familiar with skin diseases.

The general subject of Dermatology has been so associated with mysterious nomenclature that all descriptions of diseases have seemed to be meant for men who were already specialists or those peculiarly competent to investigate strange problems.

Hazen has understood this and he strips the text of unnecessary circumlocutions and tells all of us what we have wanted to know of diagnosis and treatment. It will lead the practitioner to take a deeper and better informed interest in all skin symptoms and tell him whether he can best care fo them, himself, or send them to the specialist.

The illustrations are good and comprehensive and the index and contents pages are complete.

The size of the book is not appaling and one feels that he can really use it instead of putting it on his shelves for reference when he has time to "look up" cases. He will find that it repays a complete reading.

---

LABORATORY METHODS WITH SPECIAL REFERENCE TO THE NEEDS OF THE GENERAL PRACTIONER, by B. R. G. Williams, M. D., Member of the Illinois State Medical Society, American Medical Society, etc., and E. G. C. Williams, M. D., Formerly Pathologist of Northern Michigan Hospital for the Insane, with an Intoduction by Victor C. Vaughn, M. D., LL. D., Profesor of Hygiene and Physiological Chemistry and Dean of the Department of Medicine and Surgery, University of Michigan. Third edition. Illustrated with forty-three engravings. 214 pages. St. Louis, C. V. Mosby Co., 1915. Cloth, $2.50.

This book is what it professes to be. It gives laboratory methods with reference to the General Practitioner's needs. It is by no means an epitome or a short cut to simple things while leaving the greater ones to lie in the field of the unknown and unobtainable. It does show how readily a man who is really interested in making careful diagnosis in his general work, can equip his office and become proficient in those things which, in a large community are usually referred to the Pathologist. It goes into detail as to elemental construction of a laboratory and thereby removes at once the oqjections that many a man has felt to beginning the things he knows he wants. Beyond this, it teaches tecnic clearly and concisely and will be of value in the library of the most skilled of investigators.

It does not profess to explain and use all the methods that the laboratory art has evolved, btu it does select from them those that to the Authors seem best and surest. Thus the book is kept within reasonable and convenient size.

It has rapidly gone through two editions and the increased demand has warranted this third presentation.

---

A MECHANISTIC VIEW OF WAR AND PEACE, by George W. Crile, M. D. The Macmillan Company, New York, 1915. 1.25, net.

It i ssafe to say that no book in recent years has attracted the extended attention of reviewers or has contained so much incentive to real thinking as this volume of Crile's. In the first place, Crile's name means something worth while when appended to any aticle. He is a writer who says something he knows and knows better than almost anyone else. He is a seer and a discoverer. This is true in the lay as well as in the professional mind and the result is that his book has received pages, actual pages, of review and comment in the literary journals where a few inches usually suffice.

The reason is plain when one takes his first glance at the book. In two bief pages of introduction, he startles the traditionally thinking man and the man who takes things without thinking unless he is told, into sudden activity and he says that there is a mechanical reason for war aside from the spiritual one just as there is something of the kind among animals and plants, f you please. He makes one take up the evolution theory no matter whether it has been taboo or not and begin to consider things as they are right in the trenches and on the fields and at the bases of the horrible European mess we are calling a great war. He shows the struggle for existence is something more than an acedemic phrase and he leads one to the succeeding chapters filled with eager interest in what they shall disclose.

He shows to the professional mind a new aspect of the secretions of the ductless glands and does it so nicely that one feels he must have known it all along, himself. He presents certain normal "activating patterns" that appeal instantly to the desire for graphic formulae and explain in two words the concept one has been trying to express. With those patterns in mind, the author shows their influence during normal life and then how under stress of emotion they can be changed and displaced by other patterns that take the sufferers back to the primal habits of life when fighting and constant killing were the normal and necessary parts of the day or when fleeing and great effort at protection were necessary.

The physiologic functions of these ductless glands are much better known now than they were ten years ago, but until today their primal usese had not been realized.

Crile shows the way to control them in the later stages of evolution so that their influence shall be for peace and the progress of development of the race shall be along lines that seem to this generation at least, to be good.

His book is epoch making in that in brings to the minds of men and women so recently filled with the miserable trivialities of modern problem literature in its thinly veiled appeal to the sensualities, matters which can concern them as nothing else, which will show them the truth, which will make them better parents, which will clear their thoughts and give them satisfaction in the process of thinking. The book attacks nothing, makes no quarrel, does not offend; but the thinker must be impressed with the truths it contains no matter what his previous convictions may have been. There is much that Darwin and Haeckle and Wallace and others have taught in what he says, but that is taken for granted and the applications he makes of what we really know to be so is what convinces.

He draws conclusions that must be read to be well understood. His last sentence will give a hint of the force of them and show why the entire teatise should be in the possession of every thinging man. "If the human animal were under the dominion of beings as superior to him as man is superior to domestic animals, we might expect that education would be exploited as efficiently as war has been exploited and that there might be built up a civilization freed to some extent from its menacing phylogeny."

